# Perforating necrobiosis lipoidica: good response to adalimumab^☆^^☆☆^

**DOI:** 10.1016/j.abd.2019.04.003

**Published:** 2019-11-04

**Authors:** Alexandra Brugnera Nunes de Mattos, Carolina Finardi Brummer, Gabriela Di Giunta Funchal, Daniel Holthausen Nunes

**Affiliations:** a Dermatology Service of the Hospital Universitário Polyodoro Ernani de São Thiago, Universidade Federal de Santa Catarina, Florianópolis, SC, Brazil; b Pathology and Dermatology Service of the Hospital Universitário Polyodoro Ernani de São Thiago, Department of Pathology of Universidade Federal de Santa Catarina, Florianópolis, SC, Brazil; c Dermatological Medical Residency Program in Dermatology of the Hospital Universitário Polyodoro Ernani de São Thiago, Universidade Federal de Santa Catarina, Florianópolis, SC, Brazil

Dear Editor;

Necrobiosis lipoidica (NL) is a rare granulomatous dermatosis of unknown origin, often related to diabetes mellitus (DM).^1^, ^2^ The perforating form of necrobiosis lipoidica (PNL) is even more infrequent, affecting mainly adults between 30 and 60 years, particularly women. The clinical lesion consists of coalescing plaques, of various diameters, in the classical localization of NL. The presence of keratotic “plugs,” which leave small depressions in the tissue when removed, is characteristic.^2^ Histologically, there is the elimination of the necrobiotic material through the follicular canal, in addition to the “palisade” granulomas with collagen necrobiosis.

This report details the case of a male patient, 65 years old, who referred to the appearance of papules and plaques initially in the right forearm a year ago, and three months later, in the scapular region and right leg, with pustules, edema, and erythema to proximal third of the right leg, accompanied by discreet pruritus and local discomfort. The patient has arterial hypertension and depressive disorder, and uses losartan, fluoxetine, and diosmin. He is a former smoker, while denying alcoholism and other comorbidities. The dermatological examination showed erythematous, infiltrated, annular-like plaques with ulcerated areas and fibrin on the inside of both legs, the right foot, and right arm (Fig. 1). Serological tests were negative and fasting glycemia was within normal limits. The bacilloscopy was negative for leprosy. The culture was negative for *Histoplasma capsulatum*, *Paracoccidioides brasiliensis*, and other fungi. The chest X-ray was unaltered, as were the right leg and foot X-ray. Histopathological examination revealed palisade granuloma, consisting of epithelioid histiocytes and multinucleated giant cells, centered by fibrinoid necrosis, with some neutrophils and signs of vascular damage in the dermis (Fig. 2). In the sample of the left scapular region, there was an area of epidermal perforation that communicated to the granuloma area. The search for fungi and BAAR by histochemical staining of PAS, Grocott and Ziehl-Neelsen and *M. tuberculosis* by polymerase chain reaction (PCR) were negative. The patient used rifampicin 300 mg 12/12 h and clindamycin 300 mg 12/12 h for ten weeks, presenting partial improvement of the lesions. Therefore, it was decided to start prednisone 20 mg per day, methotrexate 20 mg per week, folic acid 10 mg per week, and moisturizing dressing with calcium alginate and sodium and clobetasol daily. In two months of evolution, he presented little improvement, thus it was decided to initiate injections of adalimumab 40 mg weekly; after five months of evolution, the patient presented significant improvement of the lesions, remaining only with scars (Fig. 3).

**Figure 1:**
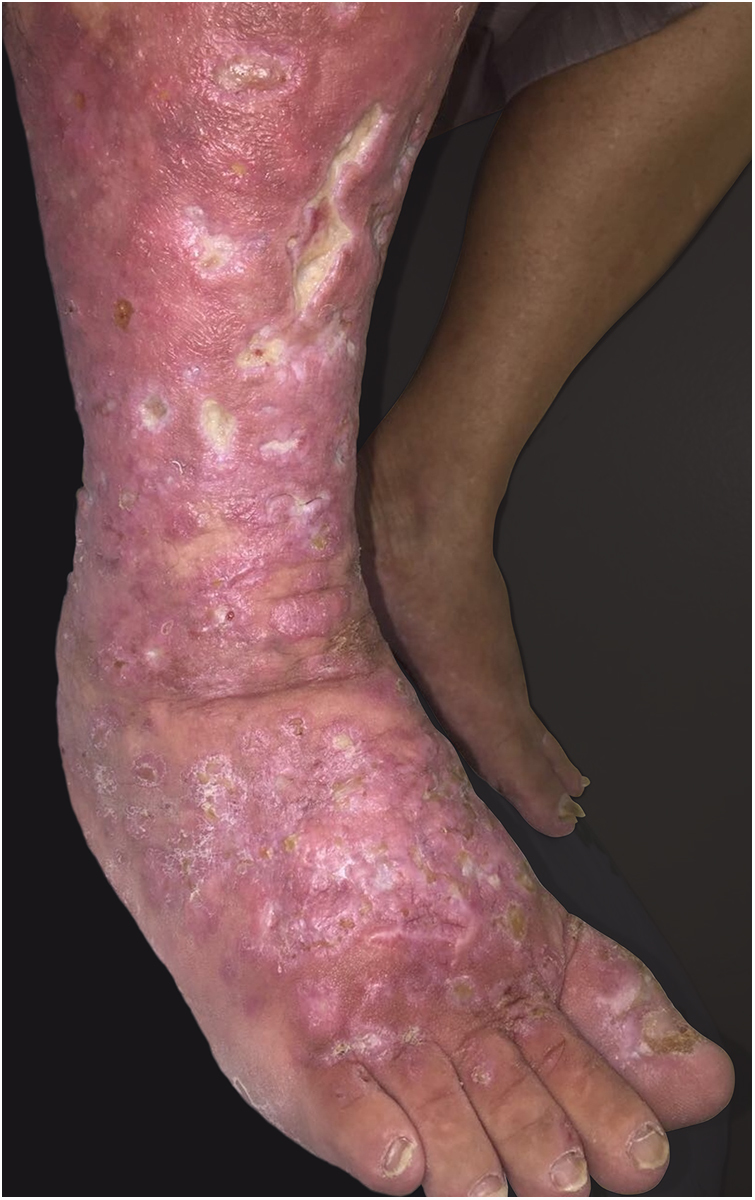
Infiltrated, ring-shaped, erythematous plaques with ulcerations, on the right leg and foot.

**Figure 2:**
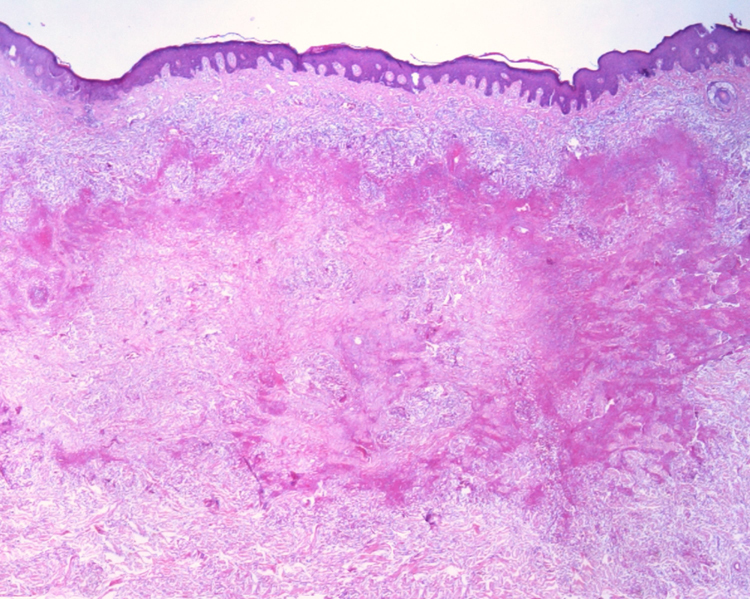
Histopathological examination shows palisade granuloma, consisting of epithelioid histiocytes and multinucleated giant cells, centered by fibrinoid necrosis, with some neutrophils and signs of vascular damage in the dermis. (Hematoxylin & eosin, ×40).

**Figure 3:**
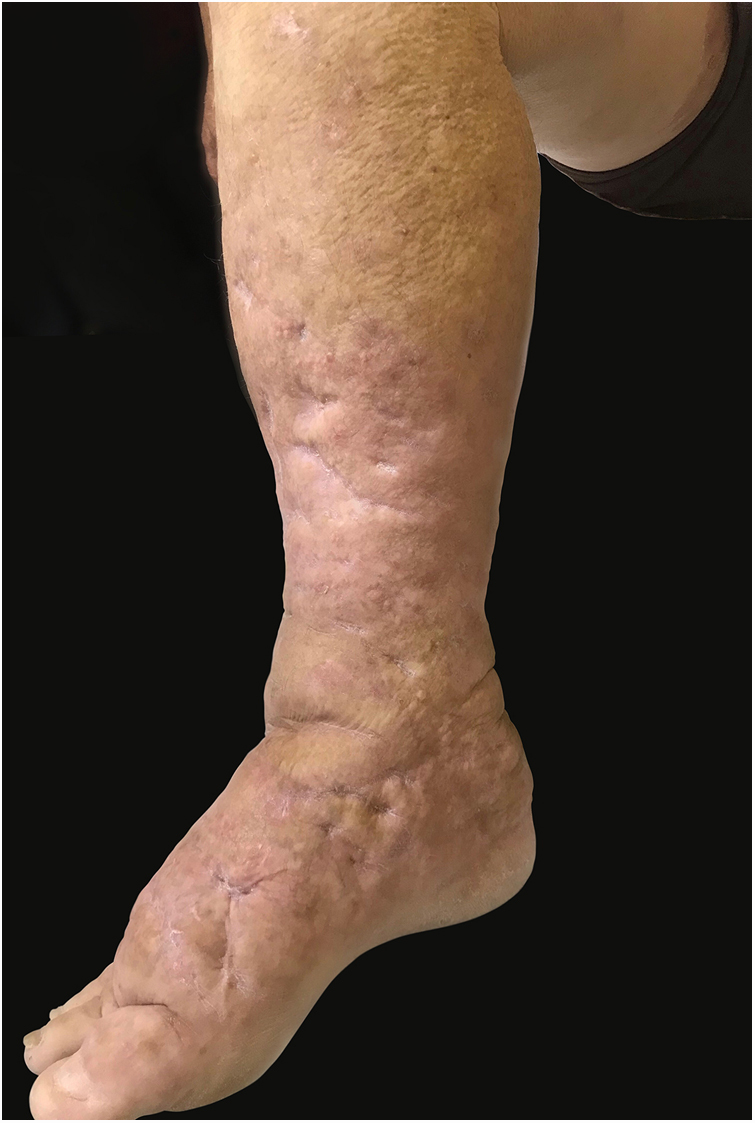
Aspect of lesions after five months of treatment with adalimumab.

The treatment of NL and PNL is difficult and often frustrating,^2^ based on reports of few cases, due to the rarity of the disease. The literature describes a broad therapeutic arsenal, ranging from local treatments such as tacrolimus, in addition to PUVA, photodynamic therapy, and systemic anti-inflammatory drugs and immunomodulators; in this context, TNF-α inhibitors show good effectiveness in controlling the formation of granulomas.^1^, ^2^ Adalimumab is a monoclonal antibody that binds to soluble TNF-α to prevent its interaction with TNF receptors on cell surfaces, thereby fixing the complement and inducing apoptosis in cells expressing TNF *in vitro*.^3^ A case study by Zhang et al. treated NL in a 29-year-old woman for 12 months using etanercept and injectable adalimumab, but they recorded more significant improvement with etanercept, with no reported side effects. Chung et al. presented a case of a rheumatoid arthritis patient who developed an NL lesion even when using adalimumab, who was treated with topical and intralesional corticosteroids and maintenance of previous medication, with improvement of the lesions.^4^ Leister et al. demonstrated the case of a 71-year-old man with NL with complete improvement after 12 weeks of adalimumab, who underwent a total period of 15 months of treatment and sustained response up to five months after the end of treatment, when the results were published.^5^ The present patient presented improvement after five months while using adalimumab without side effects up to the present moment, one and a half years after starting the medication. Based on these cases, together with this report, it is possible to understand the complex role of TNF-a in the recruitment of histiocytes and formation of granulomas, which may help to focus future prospective NL treatments on this subset of patients.

## Financial support

None declared.

## Author contribution

Alexandra Brugnera Nunes de Mattos: Composition of the manuscript; participation in the design of the study; critical review of the manuscript.

Carolina Finardi Brummer: Conception and planning of the study; composition of the manuscript; participation in the design of the study.

Gabriela Di Giunta Funchal: Critical review of the literature; critical review of the manuscript.

Daniel Holthausen Nunes: Intellectual participation in the propaedeutic and/or therapeutic conduct of the studied cases; critical review of the manuscript.

## Conflicts of interest

None declared.
